# A Case of Acute Generalized Exanthematous Pustulosis Induced by Ceftriaxone

**DOI:** 10.7759/cureus.36281

**Published:** 2023-03-17

**Authors:** Maria Inês Santos, Mafalda Sousa, Paula Cerqueira, Inês Ambrioso, Mariana Moniz Ramos

**Affiliations:** 1 Internal Medicine, Hospital Distrital de Santarém, Santarém, PRT

**Keywords:** corticoisteroids, ceftriaxone, acute generalized erythematous pustulosis, erythema, dermatosis

## Abstract

Acute generalized exanthematous pustulosis (AGEP) is a rare and severe skin disorder induced in more than 90% of cases by an adverse drug reaction. This condition is defined by an acute growth of numerous, pin-head-sized, non-follicular pustules on a background of edematous erythema that starts on the face or in the armpits and groin. It is accompanied by fever and increased inflammatory markers. We present a case of a 39-year-old male, admitted to the internal medicine department due to Streptococcus pneumoniae meningitis treated with ceftriaxone, who developed erythema covered with small sterile pustules in the face, neck, and axilla four days after antibiotic treatment. The clinical and pathological correlations confirmed the diagnosis, and the dermatosis resolved after discontinuing the drug and systemic corticosteroid treatment. Early recognition of this drug-induced dermatosis is crucial for adequate treatment.

## Introduction

Acute generalized exanthematous pustulosis (AGEP) is a rare and severe skin disorder resulting from an adverse drug reaction or a viral infection [1]. AGEP can occur in patients of any age, but it is more common in elderly patients with significant comorbidities and patients without a history of psoriasis [2]. This disease is defined by an acute growth of numerous, pin-head-sized, non-follicular pustules on a background of edematous erythema. The lesions start on the face, axilla, and groin and become more widespread. Patients may often experience other accompanying symptoms such as fever, leukocytosis with neutrophilia, and occasionally eosinophilia. Organ involvement is rare, although liver and kidney involvement has been described [3]. Symptoms resolution is observed some days after drug interruption or as a result of corticosteroid treatment.

AGEP first appeared in 1980 in France to define multiple skin lesions in several patients [4]. They set clinical criteria for diagnosing AGEP: an acute rash in individuals with no previous history of psoriasis, occurring after an infection or use of drugs, with a natural resolution [5]. It was frequently misguided as pustular psoriasis, but in 1991, a retrospective study of 63 cases of AGEP characterized the illness as having a drug etiology, thus distinguishing it from pustulous psoriasis [2]. The AGEP validation score, a standardized scoring system to help in the diagnosis, was developed by the EuroSCAR study group (RegiSCAR Project) [5]. It is based on clinical features, the course of the disease, and laboratory and histopathological findings. The scoring system ranges from 0 to 12 and classifies cases into four categories: no AGEP (<0), possible (1-4), probable (5-7), and definitive (8-12). Histological findings support the diagnosis, and two histopathology patterns are usually observed: 1) a toxic pustuloderma with spongiform intraepidermal pustulosis, a mixed upper dermal perivascular inﬂammatory inﬁltrate, and papillary edema; or 2) a leukocytoclastic vasculitis with neutrophil collections within the epidermis [5].

The etiopathogenesis is still unknown; however, it could be explained by the occasional existence of leukocytoclastic vasculitis, which evokes an Arthus-like hypersensitivity mechanism [2]. Viral causes have been reported in approximately 25% of the cases [2]. Nonetheless, pharmaceutical drugs related to treating infectious diseases have been linked to nearly 90% of the cases, among which the most critical are antibiotics. Among antibiotics, beta-lactam (24%) and macrolides (22%) are the most frequently associated with this condition, but also quinolones, aminopenicillins, sulfonamides, tetracyclines, and vancomycin have been reported [6]. Less often, it is related to other drugs, such as hydroxychloroquine, anticonvulsant medications (phenytoin and carbamazepine), antihypertensive drugs (nifedipine and atenolol), furosemide, codeine, paracetamol, acetylsalicylic acid, non-steroidal anti-inflammatories and intravenous contrast [7].

The onset of symptoms of AGEP related to antibiotic use usually occurs within the first 24 to 48 hours after the drug administration. It can be explained by the previous use of penicillin, resulting in sensibilization. However, one case series of 294 cases described a mean onset time of 9 days [8].

The treatment of AGEP centers on removing the causative drug, supportive care, infection prevention, and topical or systemic steroids. Even though most cases of GEP have a favorable prognosis with spontaneous recovery, complications like organ failure and infection can lead to a mortality rate as high as 5% [8].

## Case presentation

A 39-year-old male with a past medical history of cerebrospinal fistula of the ethmoidal bone due to trauma was admitted to the internal medicine ward due to Streptococcus pneumoniae meningitis. Within 48 hours of targeted antibiotic therapy with ceftriaxone, the patient showed clinical and laboratory improvement, with sustained apyrexia and decreased inflammatory markers.

**Figure 1:**
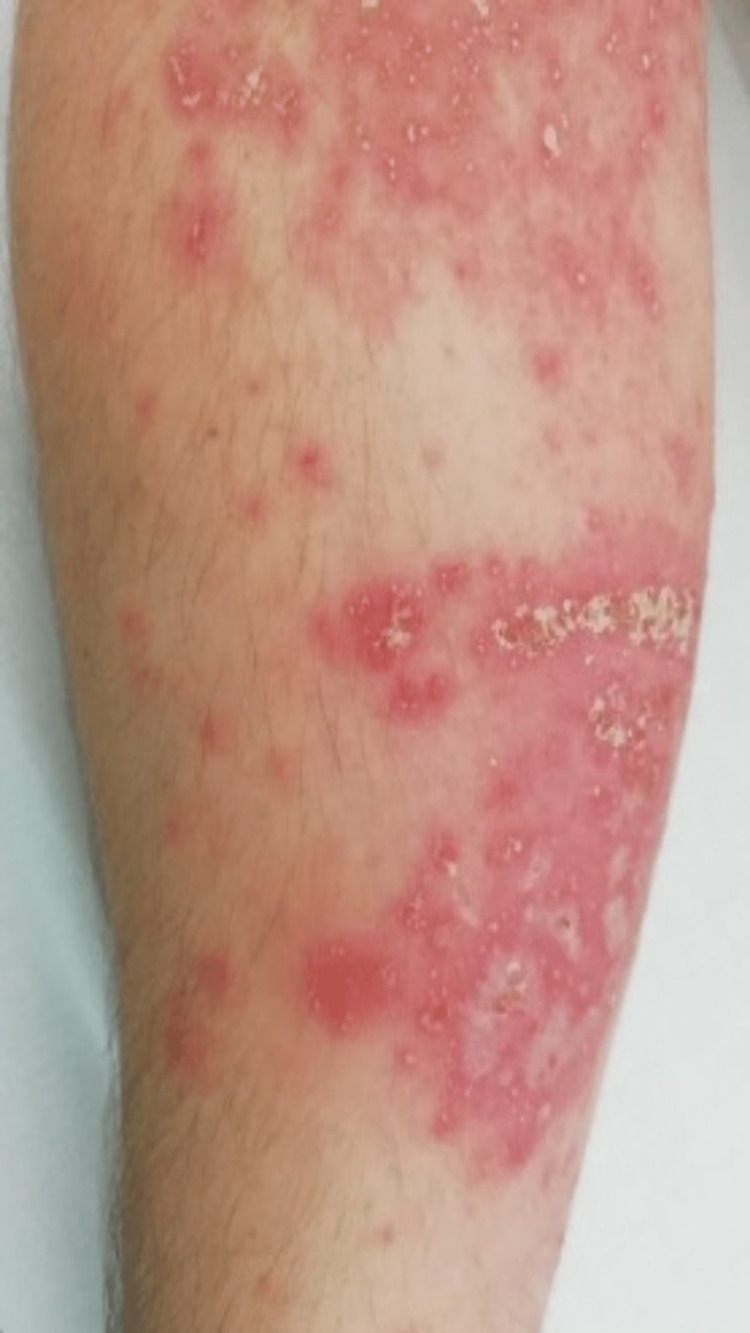
Pustular erythematous lesions on the flexion folds of the upper limbs

**Figure 2:**
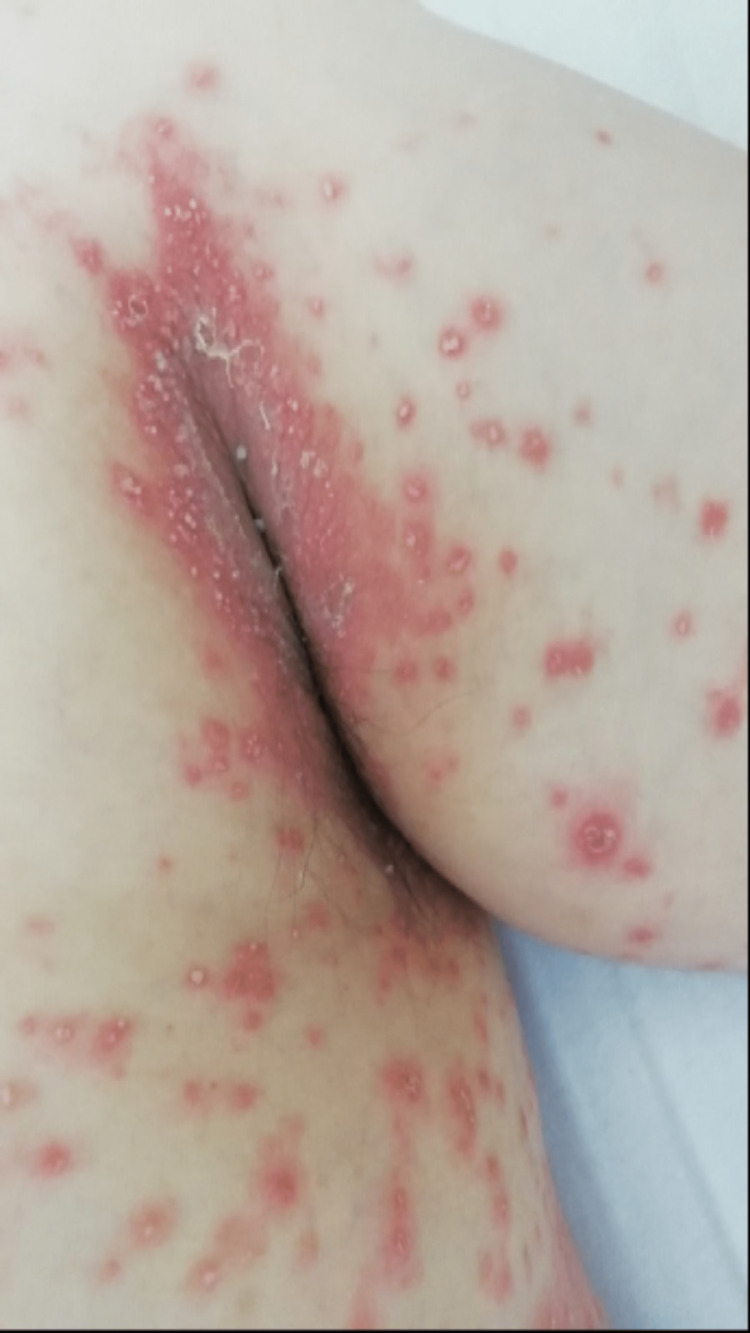
Pustular erythematous lesions of the armpits

On the fourth day of targeted antibiotic therapy, the patient presented a skin rash with pustular erythematous lesions on the flexion folds of the upper limbs (figure 1), armpits (figure 2), neck, and face (paranasal lesion). Simultaneously to the onset of the skin lesions, the patient presented fever (tympanic temperature of 38.5ºC) and a new increase in the inflammatory markers (white blood cell count of 14200/mm^3^, neutrophil count of 9100/mm^3^ and C-reactive protein of 7.05 mg/dL), without neurologic worsening. These findings were attributed to a toxidermal reaction to ceftriaxone, which was immediately suspended, and antibiotic therapy was switched to vancomycin. After examination by Dermatology, the patient was started on topical therapy with betamethasone and zinc oxide.

**Figure 3:**
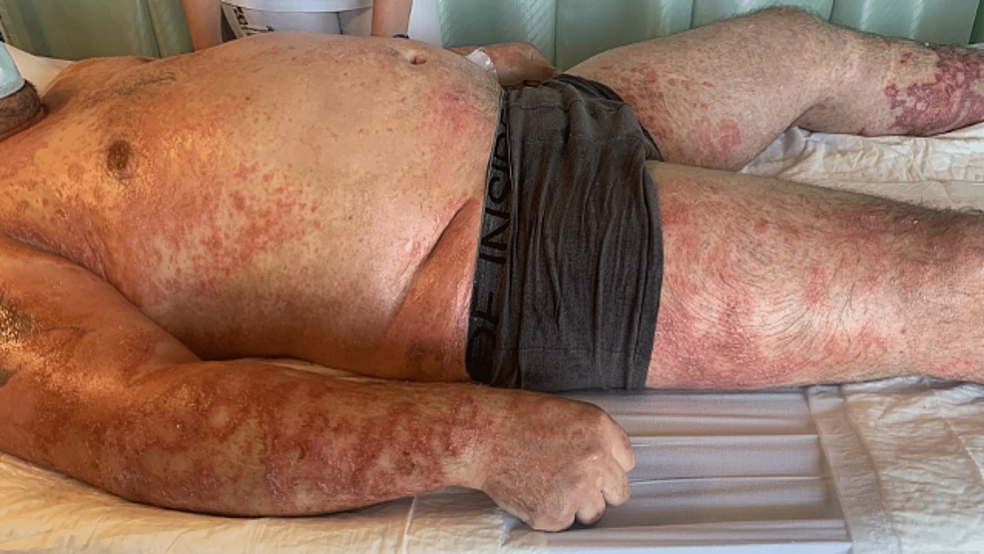
Acute generalized exanthematous pustulosis with coalescent pattern and desquamation

**Figure 4:**
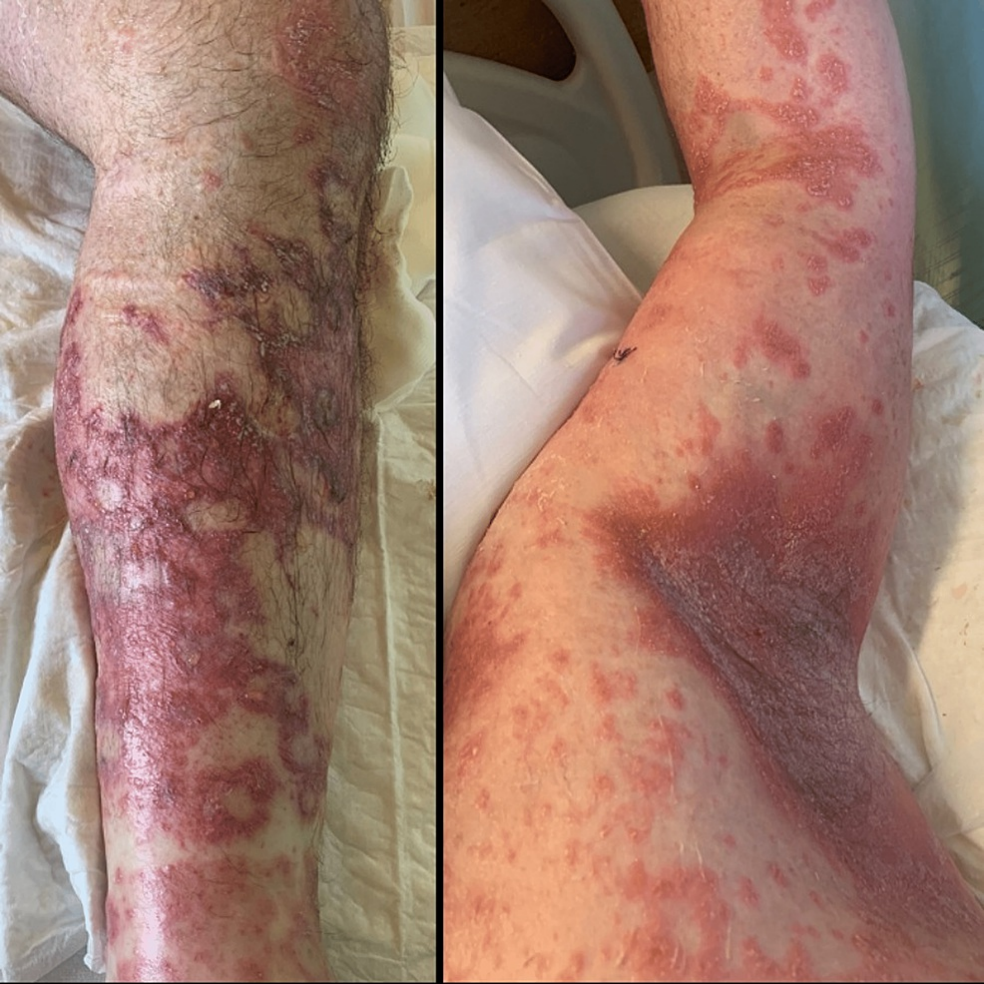
Acute generalized exanthematous pustulosis - skin lesions of the leg and armpit

**Figure 5:**
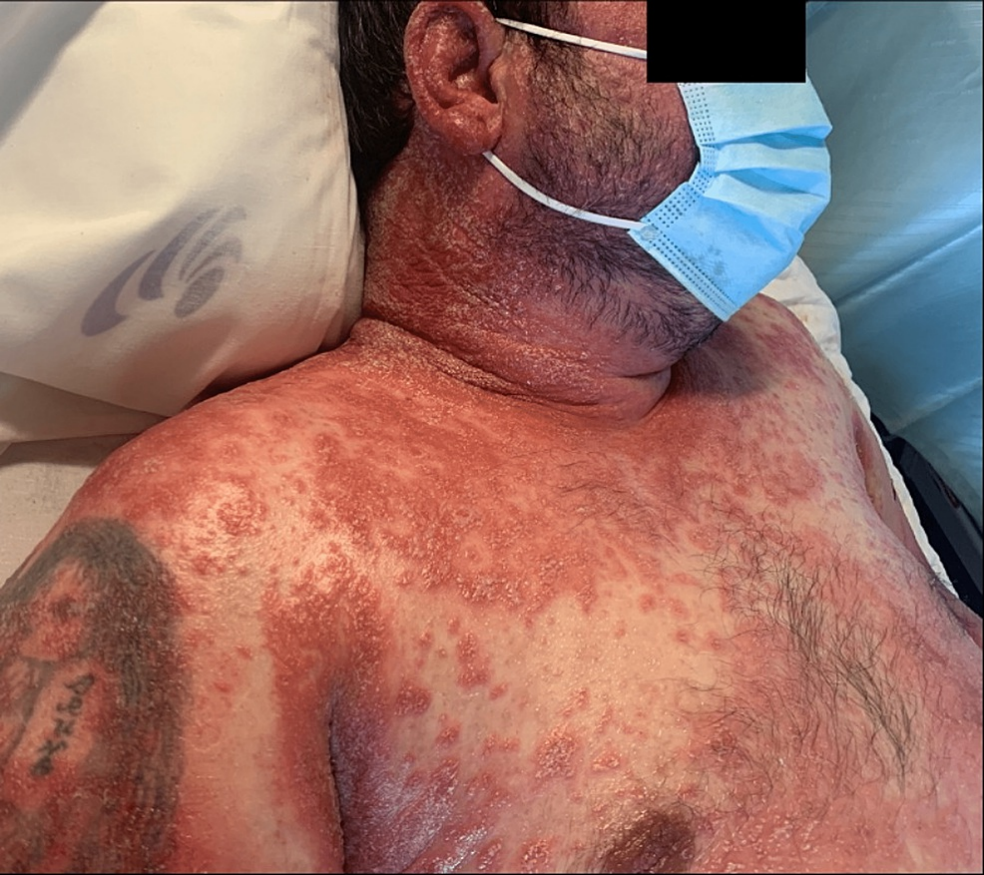
Acute generalized exanthematous pustulosis - skin lesions of the face, neck, and thorax

Despite the antibiotic switch and topical therapy, the skin lesions began to spread, showing a coalescent pattern with desquamation (figures 3-5). Due to the worsening of the skin lesions, the diagnostic hypothesis of an acute generalized exanthematous pustulosis was admitted, and a skin biopsy was performed. Treatment was started with oral corticosteroids (60mg of prednisolone daily).

**Figure 6:**
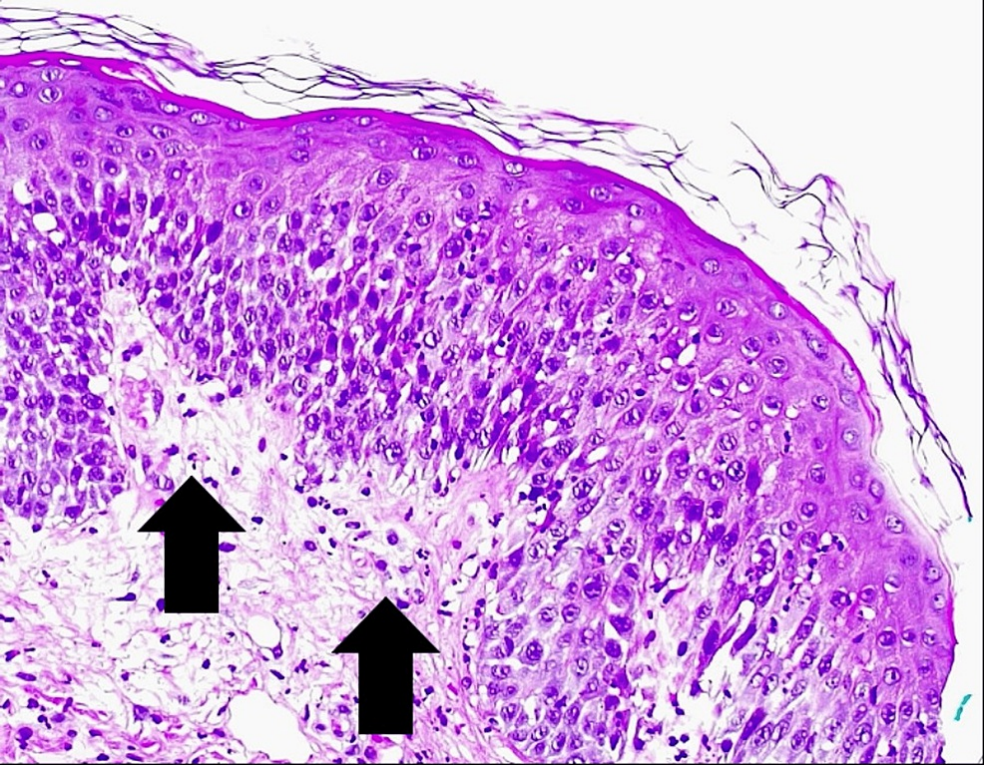
Hematoxylin and Eosin coloration (amplification 200x) - Edema and inflammatory infiltrates of the papillary dermis (arrows)

**Figure 7:**
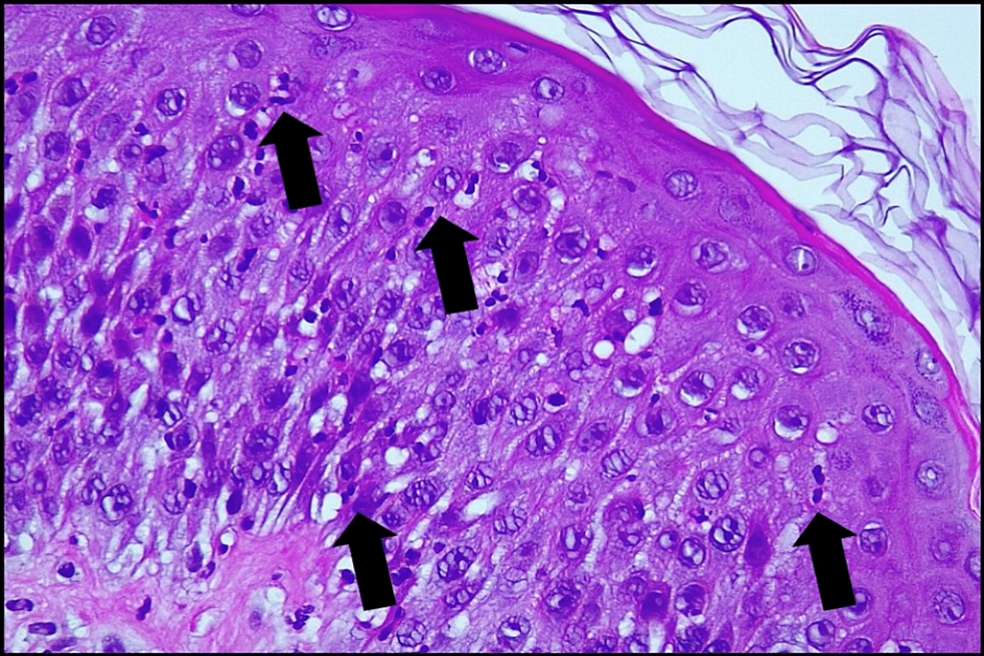
Hematoxylin and Eosin coloration (amplification 400x) - Spongiotic epidermis with exocytosis of neutrophils (arrows).

**Figure 8:**
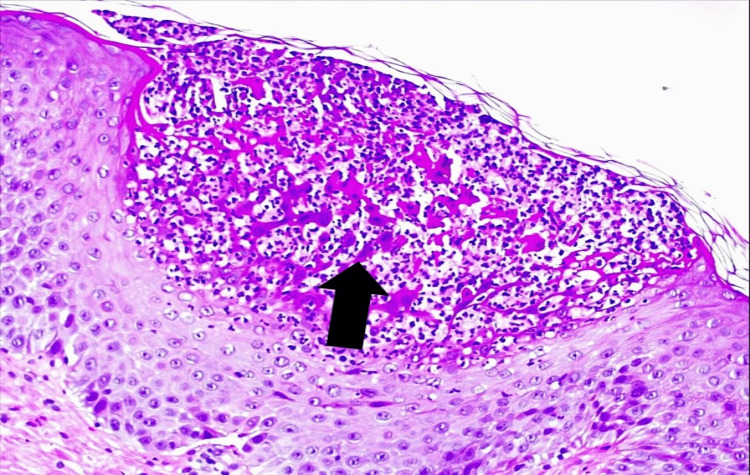
Hematoxylin and Eosin coloration (amplification 200x) - Subcorneal pustular dermatosis (arrow)

Histological examination of the skin biopsy showed a spongiotic epidermis with exocytosis of neutrophils, edema, and inflammatory infiltrates of the papillary dermis and subcorneal pustular dermatosis (figure 6-8).

**Figure 9:**
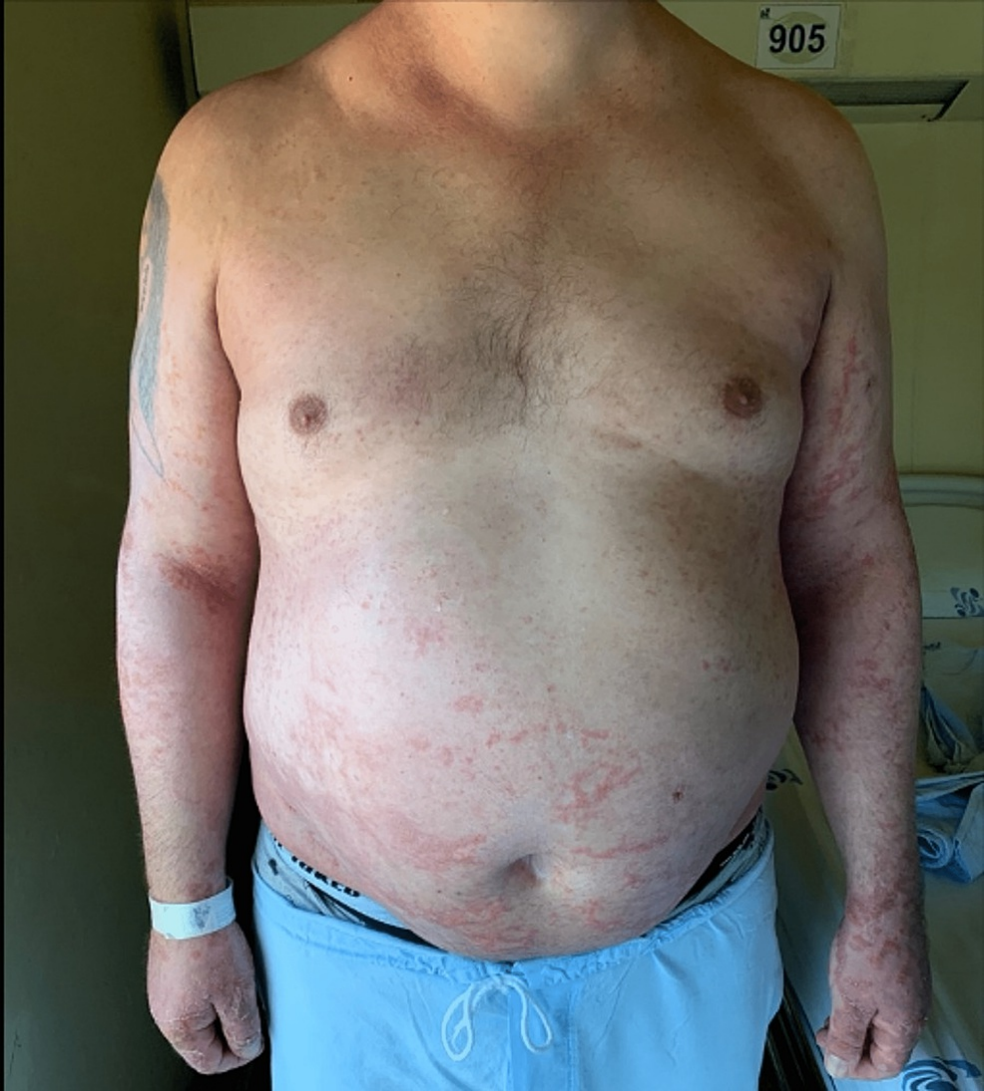
Skin lesions at discharge

**Figure 10:**
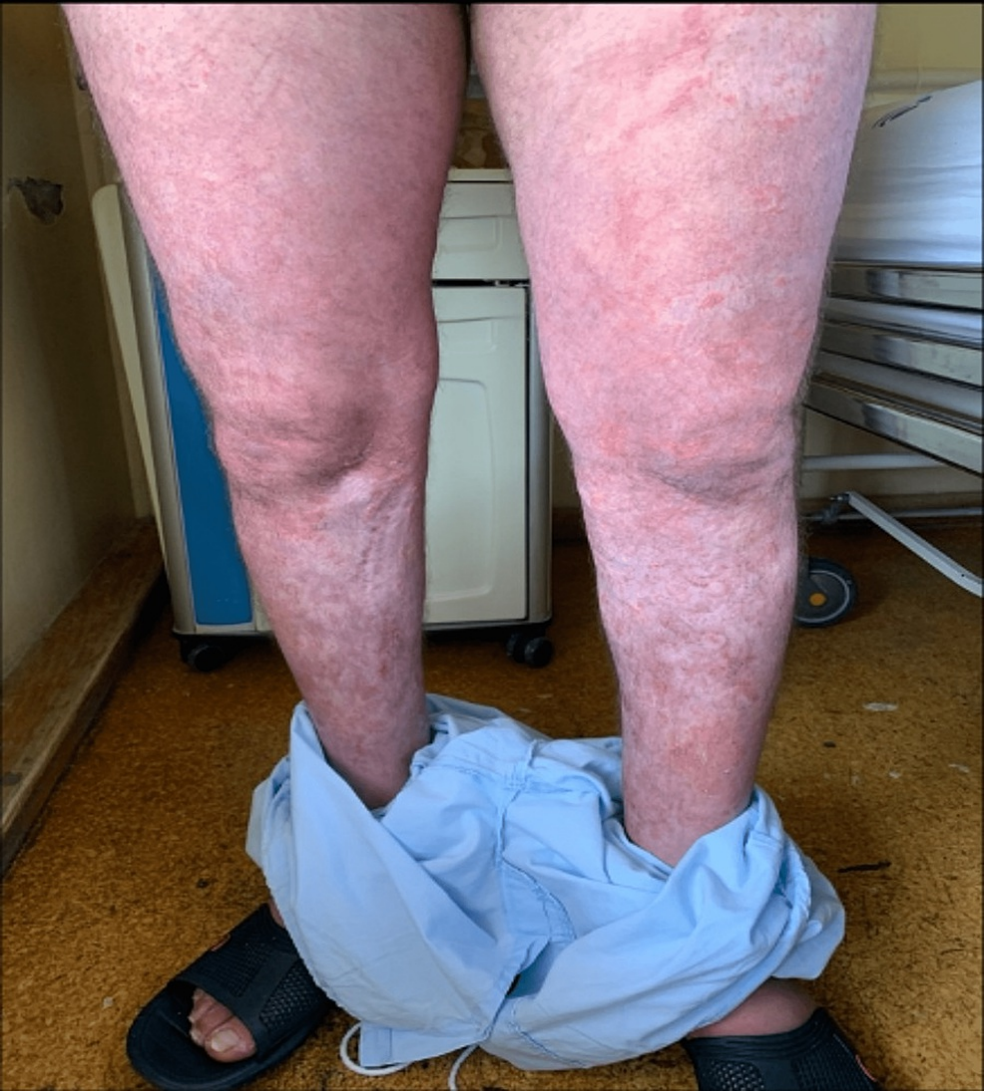
Skin lesions at discharge

These histological findings confirmed the diagnosis of acute generalized exanthematous pustulosis. After seven days of oral corticosteroids, the patient showed significant improvement in the cutaneous lesions and was discharged (figures 9, 10).

## Discussion

Acute generalized exanthematous pustulosis is extremely rare, with an estimated incidence of 1-5 per million patients yearly [9]. It is characterized by an acute onset and the spread of many sterile non-follicular pustules, generally arising on edematous erythema. Usually, patients present with fever and increasing inflammatory markers, such as leukocytosis. These lesions typically occur within 24-48 hours after antibiotics intake and resolve rapidly (within a few days after drug discontinuation) [10].

AGEP distinguishes from other drug-induced skin reactions since it manifests as pustules, easily confused with other pustular eruptions or infectious processes. Non-follicular pustules characterize AGEP, and the main differential diagnosis is pustular psoriasis. However, pustular psoriasis has a slower onset, and pustules often coalesce into extensive purulent collections [11].

Biopsy of the skin lesions is the gold standard for diagnosis [12]. Histopathology analysis presents spongiform subcorneal pustules, edematous papillary dermis with perivascular infiltrates, necrotic keratinocytes, and leukocytoclastic vasculitis with neutrophils and eosinophils [13]. AGEP is a type IV hypersensitivity reaction to drugs, but its pathologic mechanism has not been clarified. It is an inflammatory condition characterized by stimulating drug-specific T cells (cytotoxic CD4+ and CD8+ T-cells) and their migration to the skin. Once in the skin, these T-cells are activated, where they induce apoptosis of keratinocytes leading to subcorneal vesicle formation. Simultaneously, they release various proinflammatory cytokines and chemokines (particularly, chemokine (C-X-C motif) ligand 8 (CXCL8)/IL-8), leading to neutrophilic recruitment and their activation, thereby, pustule formation [14,15]. The release of cytokines also triggers systemic symptoms, such as fever, leukocytosis, and elevated C-reactive protein levels [14].

There is no specific treatment for AGEP [16]. The most important measures are discontinuing the suspected drug and supportive therapy. In severe cases, topical and systemic corticosteroids may be given, but no significant difference has been reported between treatment regimens in the course and recovery period [16,17].

In the current case, the symptoms started 96 hours after exposure to ceftriaxone, with the development of pustular erythematous skin lesions involving the flexion crease regions of the upper limbs, neck, and face. The patient improved after antibiotic discontinuation and corticosteroid treatment.

## Conclusions

The authors describe a rare case of AGEP after the administration of cephalosporin. Constitutional symptoms are frequent and systemic involvement may lead to hospitalization, although fatal complications are rare. This case report raises awareness about his disease. Early recognition and treatment with discontinuation of the causative drug are essential to the successful management of AGEP.
